# Ripening Stage and Phenolic Composition Characterization of Fruit from Different Date (*Phoenix dactylifera* L.) Cultivars in Australia

**DOI:** 10.1002/fsn3.70221

**Published:** 2025-05-03

**Authors:** Linghong Shi, Manan Sejpal, Kashif Ghafoor, Claudia Gonzalez Viejo, Sigfredo Augusto Fuentes Jara, Farhad Ahmadi, Hafiz A. R. Suleria

**Affiliations:** ^1^ School of Agriculture, Food and Ecosystem Sciences Faculty of Science, the University of Melbourne Parkville Victoria Australia; ^2^ Digital Agriculture, Food and Wine Group School of Agriculture, Food and Ecosystem Sciences, Faculty of Science, The University of Melbourne Parkville Victoria Australia

**Keywords:** Australian date palm, functional foods, phenolic profiling, phytochemicals, ripening physiology

## Abstract

We analyzed the antioxidant potential and composition of phenolic compounds in six Australian date palm cultivars (Mejhoul, Barhee, Deglet nour, Thoory, Halawi, and Khadrawy) at three ripening stages: *Kimri*, *Khalal*, and *Tamar*. Total phenolic content in date fruits at the *Kimri* stage ranged from 19.7 to 117.5 mg GAE/g, significantly exceeding that of the *Khalal* (1.22–24.4 mg GAE/g) and the *Tamar* stages (0.47–8.72 mg GAE/g). Date fruits at the *Kimri* stage had higher antioxidant potential quantified in assays such as DPPH, FRAP, ABTS, RAP, FICA, and TAC compared to those at the *Khalal* and *Tamar* stages. The LC‐ESI‐QTOF‐MS/MS analysis identified 28 phenolic compounds, categorized as 4 phenolic acids, 17 flavonoids, and 7 other phenolic compounds. Quantitative analysis using LC‐DAD suggested cultivar‐specific variations in the phenolic profile, with the Khadrawy cultivar having the highest quercetin content during the *Kimri* stage. At the *Khalal* ripening stage, Thoory and Mejhoul cultivars had higher quercetin and procyanidin A2 concentrations, respectively. At the *Tamar* stage, the Deglet nour cultivar had higher concentrations of procyanidin A2, gallic acid, and caffeic acid as compared to other date cultivars. Our findings indicate that the phenolic content decreases as date fruit matures from the *Kimri* to *Tamar* stage, with significant variations in phenolic composition and antioxidant capacity across different cultivars.

## Graphical abstract

Characterization of phenolic differences among Australian‐grown date fruit from different varieties and ripening stages. Variety differences were identified in the antioxidant capacity of date fruits. Phenolic compounds in date fruits varied with ripening stages.

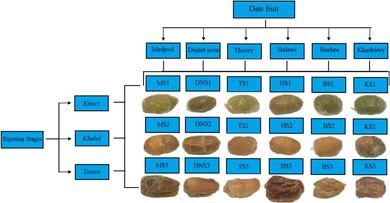

## 1 Introduction

Consumer awareness of a healthy lifestyle has driven a shift towards functional foods. This change has resulted in a transformation in consumer attitudes towards functional foods (Dhara and Nayak 2022). Bioactive compounds are naturally occurring substances found in fruits that are classified as secondary metabolites possessing potential health benefits (Uwineza and Waśkiewicz 2020). Fruits and vegetables are rich sources of bioactive compounds such as carotenoids, polyphenols, flavonoids, and tannins, which have high consumer acceptance due to their potential health benefits (Socaci et al. 2017).

Recent investigations have identified the high concentration of phenolic compounds in date palm ( *Phoenix dactylifera* L.), which are biologically active molecules with antioxidant properties and are of particular interest owing to their potential health benefits (Zihad et al. 2021). Date palm fruits are a rich source of these compounds, which may help in inhibiting oxidative stress and associated health issues (AlFaris et al. 2021; Zhang et al. 2013). Research on phenolic compounds in date palm fruit shows great potential for developing functional foods and nutraceuticals.

Ripening stage can have a large impact on the phenolic concentration in date fruits. As date fruits ripen, their chemical composition and phenolic compound concentration may change. For example, Mohamed Lemine et al. (2014) assessed six date palm cultivars from Atar and Tijigja and identified that flavonoid and total phenolic contents were greater during the *Blah* stage (first stage of maturation) compared to the *Tamar* stage (final stage of maturation). Total phenolic content averaged 728.5 mg GAE/100 g DM at the Blah stage and 558.9 mg GAE/100 g DM at the *Tamar* stage. Similarly, flavonoid content averaged 67.3 and 119.6 mg QE/100 g DM at the *Tamar* and *Blah* stages, respectively.

Cultivar is another important factor that may impact the phenolic content of date palms. Different cultivars of date palms may have unique phenolic profiles, potentially influencing their nutritional quality (Khatib et al. 2022). Identifying the differences in phenolic content among cultivars is necessary for informing agricultural practices and breeding programs to cultivate date palm cultivars with enhanced health benefits. Continued research is needed to fully understand the changes in phenolic composition and antioxidant capacity across different date palm cultivars and ripening stages. This study aimed to assess the antioxidant capacity and identify phenolic compounds in six Australian‐grown date palm cultivars at three different ripening stages, which have not been systematically researched before. Our first objective was to conduct initial assessments, including general colorimetric assays for quantification of total phenolics, flavonoids, condensed tannins, and antioxidant capacity. The second objective was characterization of phenolic compounds using LC‐ESI‐QTOF‐MS/MS and LC‐DAD analyses.

## 2 Material and Methods

### 2.1 Reagents and Chemical

Folin–Ciocalteu reagent, vanillin, gallic acid, L‐ascorbic acid, catechin, hexahydrate aluminium chloride, quercetin, DPPH, ABTS, 2,4,6‐Tris(2‐pyridyl)‐s‐triazine (TPTZ), and alizarin were obtained from Sigma‐Aldrich (Castle Hill, NSW, Australia). Sulfuric acid (98%) was purchased from RCI Labscan (Rongmuang, Thailand). Methanol, ferric chloride (Fe [III]Cl_3_•6H_2_O), acetonitrile, hydrated sodium acetate, glacial acetic acid, and hydrochloric acid were provided by Thermo Fisher Scientific Inc. (Scoresby, VIC, Australia). Sodium carbonate (anhydrous) was provided by Chem‐Supply Pty Ltd. (Adelaide, South Australia, Australia).

### 2.2 Sample Preparation

**FIGURE 1:**
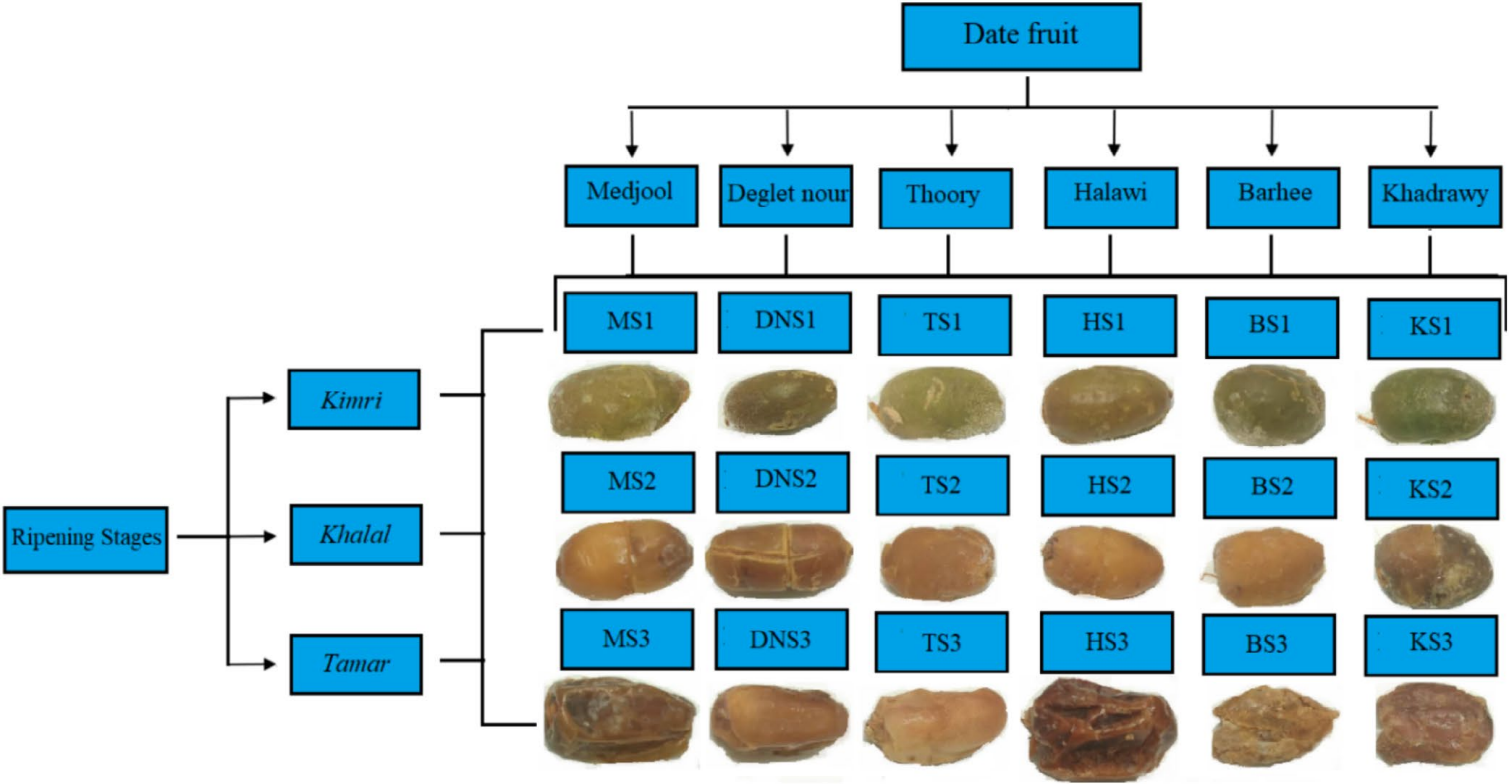
Sample abbreviations and visual appearance of date fruit cultivars of different ripening stages.

Six date cultivars, namely Mejhoul, Barhee, Deglet nour, Halawi, Thoory, and Khadrawy, were supplied from The Dessert Fruit Company (Australia), and collected in 2023 at three ripening stages: *Kimri*, *Khalal*, or *Tamar*. This resulted in a total of 18 date fruit samples. The abbreviations representing each cultivar and ripening stage, as well as the visual appearance of the date palm fruits are illustrated in Figure 1. The samples were cut and blended into a slurry using a grinder (Model 400Y, Yongkang, Zhejiang, China). Then, the samples were frozen for 12 h under −80°C and then freeze‐dried for 72 h and powdered with a grinder, and stored at −20°C.

### 2.3 Extraction Process

Extraction process was undertaken by mixing 5 g of powdered sample with 40 mL of 70% ethanol containing 0.1% formic acid. The extraction was performed using an ultrasonic cell disruptor (Branson, Digital Sonifier 450) in an ice water bath. The amplitude was set at 40%. Then, the samples were placed in a shaking incubator at 4°C and 120 rpm for 12 h. Thereafter, the extracts were subjected to centrifugation set at 8000 rpm (15 min, 4°C). The supernatant was harvested and kept at −20°C.

### 2.4 Estimation of Phenolic Compound

#### 2.4.1 Total Phenolic Content (TPC)

The analysis of TPC was made using a Folin–Ciocalteu method (Slinkard and Singleton 1977) as described in detail by Shi et al. (2024). Gallic acid was used as the standard, and the results were presented as mg gallic acid equivalents (GAE)/fresh weight.

#### 2.4.2 Total Flavonoid Content (TFC)

The AlCl_3_ colorimetric method of Christ and Müller (1960) was used for the analysis of TFC. A detailed description of the methodology is reported by Shi et al. (2024). Quercetin was used as the standard, and the results were presented as mg quercetin equivalents (QE)/fresh weight.

#### 2.4.3 Total Condensed Tannin (TCT)

The vanillin‐sulfuric acid method of Price et al. (1978) was used for measurement of TCT, as described in detail by Shi et al. (2024). Catechin was used as the standard, and the results were presented as mg catechin equivalents (CE)/fresh weight.

### 2.5 Antioxidant Activities

#### 2.5.1 2,2‐Diphenyl‐1‐Picrylhydrazyl (DPPH) Assay

The free radical scavenging activity was determined using a DPPH assay (Blois 1958), as described in detail by Shi et al. (2024). Trolox was used as the standard, and the results were presented as mg Trolox equivalents (TE)/fresh weight.

#### 2.5.2 Ferric Reducing Antioxidant Power (FRAP) Assay

The FRAP assay was performed according to the methodology of Sogi et al. (2013), with slight modifications as described by Shi et al. (2024). Trolox was used as a standard, and the results were presented as mg Trolox equivalents (TE)/fresh weight.

#### 2.5.3 Ferrous Ion Chelating Activity (FICA)

The FICA assay was undertaken following the procedure of Dinis et al. (1994), as described in detail by Shi et al. (2024). Ethylenediaminetetraacetic acid (EDTA) was used for standard curve creation, and the results were presented as mg EDTA equivalents (EE)/fresh weight.

#### 2.5.4 2,2′‐Azino‐Bis‐3‐Ethylbenzothiazoline‐6‐Sulfonic Acid (ABTS) Assay

The ABTS activity was quantified following the procedure of Re et al. (1999) with modifications as described in detail by Shi et al. (2024). Trolox was used for standard curve creation, and the results were presented as mg Trolox equivalents (TE)/fresh weight.

#### 2.5.5 Hydroxyl Radical Scavenging Activity Assay (^•^ OH‐RSA)

The ^•^OH‐RSA assay was performed following the procedure of Salgado et al. (2013) with modifications as described in detail by Shi et al. (2024). Trolox was used for standard curve creation, and the results were presented as mg Trolox equivalents (TE)/fresh weight.

#### 2.5.6 Reducing Power Assay (RPA)

The RPA assay was performed following the procedure described by Oyaizu (1986) as described in detail by Shi et al. (2024). Trolox was used for standard curve creation and the results were presented as mg Trolox equivalents (TE)/fresh weight.

#### 2.5.7 Total Antioxidant Capacity (TAC)

The TAC assay was performed following the procedure described by Prieto et al. (1999), as described in detail by Shi et al. (2024). Ascorbic acid was used for standard curve creation, and the results were presented mg ascorbic acid equivalents (AAE)/fresh weight.

### 2.6 Characterization of Phenolic Compounds

Eighteen date fruit samples were characterized for phenolic compounds using LC‐ESI‐QTOF‐MS/MS. An Agilent 1200 series HPLC system coupled with an Agilent 6530 Accurate‐Mass Quadrupole Time‐of‐Flight (Q‐TOF) LC/MS system, equipped with an electrospray ionization (ESI) source, was employed for this purpose. Detailed information regarding sample preparation, chromatographic conditions, and mass spectrometry parameters has been previously described by Shi et al. (2024) and Xie et al. (2024).

### 2.7 Quantification of Phenolic Compounds

Phenolic compound quantification was performed using an HPLC (Waters Alliance 2690, Chromatograph Separation Module) equipped with a diode array detector (Model 2998, Waters), following the methodology described by Shi et al. (2024). Calibration curves were generated using 12 standard phenolic compounds commonly found in date fruits, including gallic acid, *p*‐hydroxybenzoic acid, caffeic acid, catechin, coumaric acid, syringic acid, epicatechin, trans‐ferulic acid, procyanidin A2, sinapic acid, kaempferol, and quercetin. A heatmap was generated to visually represent the distribution and concentration of phenolic compounds across the six date fruit cultivars and three ripening stages.

### 2.8 Statistical Analysis

Data analysis was performed using one‐way analysis of variance in Minitab for Windows version 19.0 (Minitab LLC, State College, PA, USA). The Tukey's HSD test was used for mean separation at a significance threshold of *p* < 0.05. Pearson's correlation analysis was conducted to assess the relationships between phenolic content (TPC, TCT, TFC) and the antioxidant assays (DPPH, FRAP, ABTS, •OH‐RSA, RAP, FICA, and TAC). The PCA was also employed to establish correlations between antioxidant assays and phenolic content across 6 date cultivars at 3 ripening stages.

## 3 Results and Discussion

### 3.1 Phenolic Content Estimation (TPC, TFC, and TCT)

The data of TPC, TFC, and TCT from date fruit cultivars at three ripening stages is reported in Table 1. The highest TPC was quantified at the *Kimri* stage, followed by *Khalal* and *Tamar* stages. Also, significant cultivar differences in TPC were observed (*p* < 0.05). Supporting this, Mohamed et al. (2014) reported the impact of cultivar differences on TPC content in date fruits.

**TABLE 1:** Total phenolics, flavonoids, and condensed tannin contents, and antioxidant activity of fruits from different date cultivars and ripening stages (mean ± standard deviation, *n* = 3).

Items	Date cultivar					
	Mejhoul	Deglet nour	Thoory	Halawi	Barhee	Khadrawy
*Kimri* stage	MS1	DNS1	TS1	HS1	BS1	KS1
TPC (mg GAE/g)	42.1 ± 1.77^c^ *	98.8 ± 7.91^b^ *	117.5 ± 8.62^a^ *	33.1 ± 2.57^d^ *	19.7 ± 1.49^e^ *	48.9 ± 3.65^c^ *
TCT (mg CE/g)	ND	0.26 ± 0.20*	0.15 ± 0.12*	ND	ND	ND
TFC (mg QE/g)	0.63 ± 0.05^c^ *	0.96 ± 0.07^a^ *	0.78 ± 0.05^b^ *	0.25 ± 0.04^e^ *	0.08 ± 0.01^f^ *	0.41 ± 0.03^d^ *
DPPH (mg TE/g)	9.66 ± 0.09^e^	38.9 ± 1.23^b^ *	42.4 ± 0.69^a^ *	8.98 ± 0.29^e^ *	14.3 ± 0.60^d^ *	33.2 ± 1.09^c^ *
FRAP (mg TE/g)	49.4 ± 0.22^cd^ *	64.1 ± 0.12^b^ *	63.9 ± 0.10^b^ *	47.8 ± 0.82^cd^ *	42.0 ± 3.61^d^ *	81.7 ± 11.8^a^ *
ABTS (mg AAE/g)	23.7 ± 1.81^c^ *	15.7 ± 0.10^e^ *	62.5 ± 0.18^a^ *	19.0 ± 2.23^d^ *	23.3 ± 0.05^c^ *	31.3 ± 0.37^b^ *
^•^OH^−^RSA (mg TE/g)	1.90 ± 0.11^bc^	1.32 ± 0.11^d^	1.33 ± 0.05^d^	2.37 ± 0.06^a^	2.60 ± 0.09^a^	1.74 ± 0.15^c^
RAP (mg TE/g)	21.3 ± 2.34^c^ *	35.4 ± 2.61^a^ *	26.9 ± 1.17^b^ *	17.2 ± 0.35^cd^ *	14.9 ± 0.50^d^ *	27.7 ± 0.95^b^ *
FICA (mg EE/g)	2.34 ± 0.25^ab^ *	2.57 ± 0.21^a^	2.36 ± 0.18 ^a^ *	2.43 ± 0.38^ab^ *	2.07 ± 0.20^b^	2.09 ± 0.21^b^ *
TAC (mg AAE/g)	35.0 ± 2.15^c^ *	49.1 ± 1.45^a^ *	44.8 ± 1.22^b^ *	21.0 ± 0.29^d^ *	13.0 ± 1.24^e^ *	37.1 ± 2.19^c^ *

*Khalal* stage	MS2	DNS2	TS2	HS2	BS2	KS2
TPC (mg GAE/g)	10.1 ± 0.17^b^	24.4 ± 1.12^a^	24.1 ± 2.03^a^	7.53 ± 0.55^c^	1.88 ± 0.05^d^	1.22 ± 0.06^d^
TCT (mg CE/g)	ND	ND	ND	ND	ND	ND
TFC (mg QE/g)	ND	ND	ND	ND	ND	ND
DPPH (mg TE/g)	10.5 ± 0.10^c^ *	21.8 ± 0.67^a^	18.2 ± 0.62^b^	8.20 ± 0.14^d^	5.40 ± 0.31^e^	5.16 ± 0.64^e^
FRAP (mg TE/g)	26.6 ± 3.28^c^	55.3 ± 3.12^a^	45.0 ± 2.48^b^	13.9 ± 1.09^d^	4.33 ± 0.22^e^	2.33 ± 0.17^e^
ABTS (mg AAE/g)	14.6 ± 0.32^b^	15.4 ± 0.53^b^	18.3 ± 0.56^a^	7.82 ± 0.97^c^	3.97 ± 0.08^d^	15.37 ± 0.30^b^
^•^OH^−^RSA (mg TE/g)	3.02 ± 0.18^b^	2.45 ± 0.08^c^	2.49 ± 0.04^c^	3.23 ± 0.05^b^	3.51 ± 0.02^a^	3.58 ± 0.01^a^ *
RAP (mg TE/g)	4.15 ± 2.10^c^	29.4 ± 0.67^a^	11.0 ± 1.20^b^	4.61 ± 0.67^c^	ND	ND
FICA (mg EE/g)	2.27 ± 0.32^a^	2.13 ± 0.28^ab^	2.32 ± 0.33 ^a^	2.26 ± 0.05^a^	2.45 ± 0.05^a^ *	1.99 ± 0.26^b^
TAC (mg AAE/g)	11.6 ± 0.59^d^	28.0 ± 0.69^a^	22.9 ± 1.12^b^	15.3 ± 0.30^c^	11.2 ± 0.77^d^	2.68 ± 0.20^e^

*Tamar* stage	MS3	DNS3	TS3	HS3	BS3	KS3
TPC (mg GAE/g)	8.72 ± 0.07^a^	1.30 ± 0.06^c^	0.47 ± 0.04^d^	1.74 ± 0.03^b^	0.59 ± 0.04^d^	1.71 ± 0.14^b^
TCT (mg CE/g)	ND	ND	ND	ND	ND	ND
TFC (mg QE/g)	ND	ND	ND	ND	ND	ND
DPPH (mg TE/g)	5.41 ± 0.79^a^	3.71 ± 0.36^b^	5.24 ± 0.66^ab^	5.26 ± 0.49^ab^	4.67 ± 0.66^ab^	4.61 ± 0.35^ab^
FRAP (mg TE/g)	1.34 ± 0.08^b^	2.11 ± 0.25^a^	2.37 ± 0.16^a^	0.71 ± 0.04^c^	1.42 ± 0.04^b^	1.66 ± 0.09^b^
ABTS (mg AAE/g)	0.79 ± 0.08^c^	2.41 ± 0.29^b^	2.09 ± 0.19^b^	1.39 ± 0.07^c^	7.35 ± 0.40^a^	7.83 ± 0.16^a^
^•^OH‐RSA (mg TE/g)	3.74 ± 0.01^a^ *	3.58 ± 0.02^cd^ *	3.61 ± 0.01^bc^ *	3.54 ± 0.02^de^ *	3.53 ± 0.01^de^ *	3.51 ± 0.04^e^
RAP (mg TE/g)	ND	ND	ND	ND	ND	ND
FICA (mg EE/g)	2.08 ± 0.33^ab^	2.63 ± 0.26^a^ *	1.71 ± 0.36^b^	1.75 ± 0.18^b^	1.70 ± 0.08^b^	1.85 ± 0.11^b^
TAC (mg AAE/g)	13.1 ± 0.07^a^	9.62 ± 1.40^bc^	11.1 ± 0.22^ab^	10.4 ± 0.10^bc^	7.93 ± 0.50^d^	9.22 ± 1.21^bcd^

*Note:* superscript values indicate mean in a row with a significant difference (*p* < 0.05; Tukey's test).

Abbreviations: AAE, ascorbic acid equivalents; CE, catechin equivalents; EE, EDTA equivalents; GAE, gallic acid equivalents; ND, not detectedQE, quercetin equivalents; TE, Trolox equivalents.

* The highest value among the three ripening stages. Sample abbreviations were defined in Figure 1.

The TPC decreased significantly from the *Kimri* to the *Tamar* stage across all date cultivars. The TPC value at the *Kimri* stage was the highest in TS1 and the lowest in BS1. The TPC value in the *Khalal* stage was highest in both TS2 and DNS2 date cultivars. The TPC ranged from 8.72 to 42.07 mg GAE/g in Mejhoul, 1.30 to 98.77 mg GAE/g in Deglet nour, 0.47 to 117.46 mg GAE/g in Thoory, 1.74 to 33.1 mg GAE/g in Halawi, 0.59 to 19.68 mg GAE/g in Barhee, and 1.71 to 48.9 mg GAE/g in Khadrawy. The lowest TPC (0.47 mg GAE/g) was observed in the Thoory cultivar at the *Tamar* stage. Previous studies have reported that as date fruit matures, TPC decreases mainly because of oxidation by polyphenol oxidase (Bano et al. 2022). Shahdadi et al. (2015) reported that TPC decreased with the progressive maturation stages in date fruit. In their study, the TPC in two cultivars (Mozafati and Kalute) declined from around 11 and 9 mg GAE/g at the *Khalal* stage to around 8 and 7 mg GAE/1 g at *Tamar* stage, respectively. Mohamed Lemine et al. (2014) also observed a similar trend, identifying that the TPC at the *Khalal* stage averaged 7.28 mg GAE/g, significantly higher than the mean value of 5.59 mg GAE/g at the *Tamar* stage. Rashidinejad and Ahmmed (2024) also reported that the TPC in damson plums decreased with maturation.

Similar trends were also observed for TFC and TCT. At the *Kimri* stage, TFC and TCT ranged from 0.08 to 0.96 mg QE/g and 0.15 to 0.26 mg CE/g, respectively. However, flavonoids and condensed tannins were not detected at the *Khalal* and *Tamar* stages. The trend observed in TFC is consistent with previous studies by Bano et al. (2022) and Amira et al. (2012). Mohamed et al. (2014) reported a higher TFC (1.74–3.39 mg CE/100 g) in Sudanese date cultivars compared to date fruits at the *Tamar* stage in this study. These discrepancies may be attributed to factors such as extraction methods, geographical origin, harvest time, cultivar type, and environmental conditions. The TCT concentration also exhibited a decreasing trend as the fruit ripened. This reduction in tannins contributes to the less astringent taste and increased palatability of ripe fruit (Souli et al. 2018).

### 3.2 Antioxidant Estimation

The DPPH free radical scavenging activity varied significantly across cultivars and ripening stages. At the *Kimri* stage, the Thoory cultivar had the highest activity (42.35 mg TE/g). However, the scavenging activity was the lowest in the Mejhoul cultivar (9.66 mg TE/g). Similarly, the Deglet nour cultivar had the highest activity at the *Khalal* stage (21.76 mg TE/g). At the *Tamar* stage, the Mejhoul and Deglet nour cultivars had the highest and lowest activity of 5.41 and 3.71 mg TE/g, respectively. The decrease in DPPH activity with fruit maturation is similar to the trend in TPC and is consistent with previous research on date palm fruits (Amira et al. 2012; Bano et al. 2022; Haider et al. 2018; Mohamed et al. 2014). Variations in antioxidant activity among cultivars may be attributed to factors such as agricultural practices, soil type, and temperature. In contrast, the changes in DPPH activity among ripening stages may likely be due to the reduction in enzymatic activity as the fruit ripens (Bano et al. 2022).

The FRAP assay quantifies the antioxidant activity through reducing ferric Fe^3+^ to ferrous Fe^2+^. This assay evaluates the reducing power, rather than its direct free radical scavenging ability (Jdaini et al. 2023). Khadrawy cultivar had the highest FRAP at the *Kimri* stage (81.7 mg TE/g); however, Deglet nour cultivar had the highest reducing power at the *Khalal* stage (55.3 mg TE/g). At the *Tamar* stage, Thoory cultivar had the highest FRAP (2.37 mg TE/g). Supporting this observation, Haider et al. (2018) also reported a decrease in FRAP activity as the fruit maturity advanced from *Kimri* to *Tamar* stage.

In this study, Thoory cultivar had the highest ABTS at the *Kimri* and *Khalal* stages, averaging 62.5 and 18.3 mg AAE/g, respectively. Deglet nour cultivar had the highest ABTS at the *Tamar* stage (2.41 mg AAE/g). Overall, the ABTS values among date fruit cultivars at different ripening stages ranged from 0.79 to 62.5 mg AAE/g, showing a downward trend with date fruit ripening. In support of this finding, Rashidinejad and Ahmmed (2024) reported that the ABTS value in damson plums decreased as the fruit ripened, declining from approximately 6.60 μmol TE/g to 5.90 μmol TE/g.

Among date fruit cultivars, Deglet nour had the highest RPA at the *Kimri* stage (35.4 TE/g), with a significant decrease in RPA with maturation (Table 1). Barhee and Khadrawy cultivars showed a complete reduction of RPA values to zero at this stage. At the *Tamar* stage, all six cultivars showed no reducing power. Contrary to this observation, Amira et al. (2012) reported a decrease in antioxidant capacity during ripening due to enzymatic factors or environmental conditions.

The •OH‐RSA assay evaluates the capacity of a sample to neutralize hydroxyl radicals, highly reactive species implicated in oxidative stress. By assessing the ability of a sample to counteract these radicals, this assay provides information about its potential antioxidant properties and associated health benefits (Kutlu et al. 2014). Among all the cultivars and ripening stages, the highest •OH‐RSA value was quantified at the *Tamar* stage, with Mejhoul cultivar having the highest value of 3.74 mg TE/g. Conversely, the lowest value was quantified at the *Kimri* stage. Contrary to other antioxidant assays, antioxidant capacity quantified using the •OH‐RSA assay increased with fruit ripening from the *Kimri* to the *Tamar* stage.

Deglet nour cultivar at the *Kimri* stage had the highest FICA and TAC values (2.57 mg EE/g and 49.1 mg AAE/g, respectively). However, Halawi cultivar at the *Tamar* stage had the lowest values (1.75 mg EE/g and 2.68 mg AAE/g, respectively).

Overall, the results of most antioxidant assays agree with the observation that the antioxidant capacity of date fruits tends to decrease with ripening. However, contrasting trends were observed with •OH‐RSA and FICA assays, with •OH‐RSA increasing and FICA showing no significant change with ripening. Further investigation is needed to determine whether this discrepancy is due to the underlying biological factors related to cultivar differences.

### 3.3 Correlation Analysis

As presented in Table 2, TPC was correlated positively with TFC, TCT, DPPH, FRAP, ABTS, and TAC (*r* = 0.923, 0.886, 0.895, 0.760, 0.798 and 0.912, respectively; *p* < 0.05). A significant negative correlation existed between TPC and ^•^OH‐RSA (*r* = −0.909, *p* < 0.05). However, the correlation between FICA and TPC was not significant (*r* = 0.474, *p* < 0.05). A similar trend was observed among different phenolic and antioxidant assays. Overall, the correlation was significantly positively correlated and in line with the previous studies, reporting that flavonoids contributed less to the total antioxidant capacity and that the correlation between FICA and phenolic, condensed tannin, and flavonoid content was insignificant (Shi et al. 2023; Suleria et al. 2020).

**TABLE 2:** Pearson's correlation between antioxidant capacity assessed using different assays.

Variables	TPC	TFC	TCT	DPPH	FRAP	ABTS	^•^OH^−^RSA	RPA	FICA
TFC	0.923**								
TCT	0.886**	0.813**							
DPPH	0.895**	0.855**	0.796**						
FRAP	0.760**	0.860**	0.697*	0.884**					
ABTS	0.798**	0.769**	0.601*	0.823**	0.810*				
^•^OH‐RSA	−0.909*	−0.947*	−0.853*	−0.886*	−0.928*	−0.813*			
RPA	0.824**	0.865**	0.821**	0.833**	0.901**	0.690*	−0.951**		
FICA	0.474	0.482	0.533	0.367	0.399	0.304	−0.470	0.415	
TAC	0.912**	0.935**	0.856**	0.893**	0.875**	0.723**	−0.951**	0.921**	0.442

** Significant correlation at *p ≤* 0.01.

* Significant correlation at *p ≤* 0.05.

**FIGURE 2:**
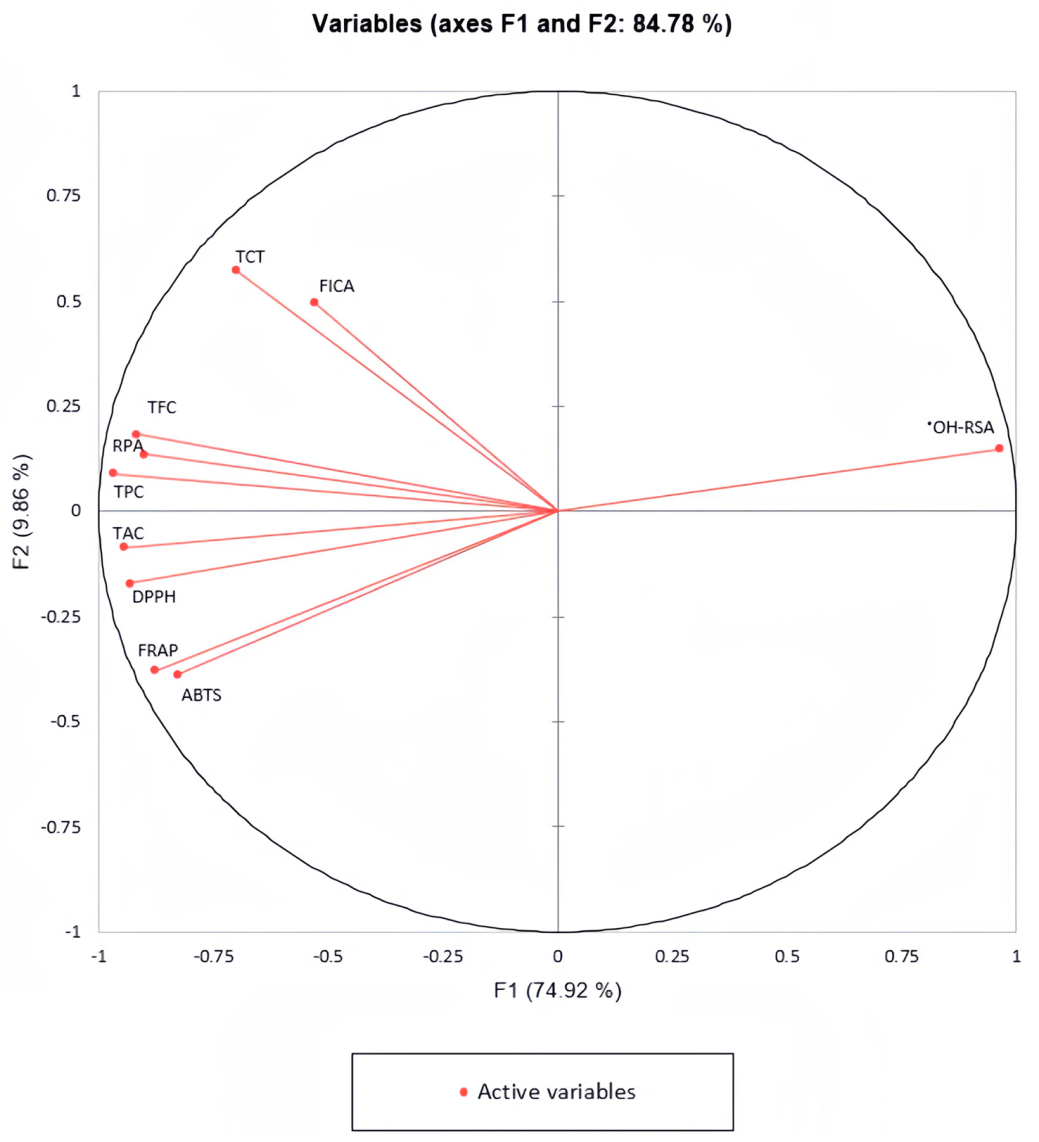
Principal component analysis of phenolic content (TPC, TFC, and TCT) and antioxidant activities (FICA, ^•^OH‐RSA, RPA, DPPH, ABTS, FRAP and TAC) from six date fruit cultivars at three ripening stages.

The PCA chart showed that the first two components (F1 and F2) explained 84.78% of the variability of the initial data (Figure 2). After evaluating the data, we found that antioxidant assays (RPA, TAC, DPPS, FRAP, and ABTS) were highly related to TPC and TFC while there is a higher positive correlation between FICA and TCT.

The evaluation of the data shows that the antioxidant activity of date palm extract may also be influenced by non‐phenolic factors. While most antioxidant assays exhibit a positive correlation with the concentration of phenolic compounds. How ever, assay like hydroxyl radical scavenging activity (•OH‐RSA), demonstrate opposite results. This indicates the potential presence of other components in dates that could affect hydroxyl radical scavenging ability. However, the overall trend still indicates that higher phenolic content corresponds to higher antioxidant capacity.

### 3.4 Characterization of Phenolic Compounds

The LC‐ESIQTOF‐MS/MS analysis in both positive and negative ionization modes identified a total of 28 different phenolic compounds across all 18 samples (Table 3). This characterization included 4 phenolic acids, 17 flavonoids, and 7 other phenolic compounds.

**TABLE 3:** Characterization of phenolic compounds in date fruits by LC‐ESI‐QTOF‐MS/MS.

No.	Proposed compounds	Molecular formula	RT (min)	Ionization (ESI+/ESI−)	Molecular weight	Theoretical (m/z)	Observed (m/z)	Error (ppm)	MS/MS product ion	Sample
**Phenolic acids** **Hydroxybenzoic acids**										
1	3,4‐*O*‐Dimethylgallic acid	C_9_H_10_O_5_	12.447	[M + H]^+^	198.0544	199.0617	199.0609	−4.0	153, 139, 125, 111	HS2*
2	4‐*O*‐Methylgallic acid	C_8_H_8_O_5_	51.955	[M + H]^+^	184.0357	185.043	185.0422	−4.3	170, 142	MS2*, DNS1, KS2, BS2, DNS2
**Hydroxycinnamic acids**										
3	5–5’‐Dehydrodiferulic acid	C_20_H_18_O_8_	56.910	[M + H]^+^	386.0974	387.1047	387.1066	4.9	369	TS3*, TS2
**Hydroxyphenylpentanoic acids**										
4	5‐(3′‐Methoxy‐4′‐hydroxyphenyl)‐γ‐valerolactone	C_12_H_14_O_4_	56.910	[M + H]^+^	222.0885	223.0958	223.0957	−0.4	205	HS1*, KS3
**Flavonoids** **Anthocyanins**										
5	4‐*O*‐Methyldelphinidin 3‐*O*‐D‐glucoside	C_22_H_23_O_12_	54.929	[M + H]^+^	479.1219	480.1292	480.1276	−3.3	317, 302, 285, 271	TS1*
6	Cyanidin 3‐*O*‐(2‐*O*‐(6‐*O*‐(E)‐caffeoyl‐D glucoside)‐D‐glucoside)‐5‐*O*‐D‐glucoside	C_43_H_49_O_24_	62.291	[M + H]^+^	949.2569	950.2642	950.2677	3.7	162, 324, 514	DNS2*
**Dihydrochalcones**										
7	3‐Hydroxyphloretin 2’‐*O*‐xylosyl‐glucoside	C_26_H_32_O_15_	43.996	[M‐H]^−^	584.1767	583.1694	583.1697	0.5	289	TS2*, KS1, TS3
**Flavanols**										
8	4”‐*O*‐Methylepigallocatechin 3‐O‐gallate	C_23_H_20_O_11_	12.231	[M + H]^+^	320.0915	321.0988	321.0974	−4.4	169, 319	HS2*, TS1, KS2, BS2
9	4’‐*O*‐Methylepigallocatechin	C_16_H_16_O_7_	12.716	[M + H]^+^	472.0992	471.0919	471.0907	−2.5	92, 121	DNS3*
**Flavanones**										
10	8‐Prenylnaringenin	C_20_H_20_O_5_	57.621	[M + H]^+^	340.1302	341.1375	341.1373	−0.6	323, 271, 137	HS3*
**Flavones**										
11	Apigenin 7‐*O*‐apiosyl‐glucoside	C_26_H_28_O_14_	11.885	[M + H]^+^	564.1456	565.1529	565.1548	3.4	296	HS3*
12	Apigenin 7‐*O*‐glucuronide	C_21_H_18_O_11_	12.931	[M + H]^+^	446.0829	447.0902	447.0922	4.5	271, 253	BS3*, HS3
13	Luteolin 7‐*O*‐(2‐apiosyl‐glucoside)	C_26_H_28_O_15_	58.253	[M + H]^+^	580.1459	581.1532	581.1557	4.3	180, 324	TS3*, MS1, BS1, DNS1, TS1, HS1, KS2, TS2, KS3, BS3
14	Gardenin B	C_19_H_18_O_7_	58.839	[M + H]^+^	358.1054	359.1127	359.1132	1.4	344, 329, 311	DNS1*
15	Chrysoeriol 7‐*O*‐(6″‐malonyl‐apiosyl‐glucoside)	C_30_H_32_O_18_	57.032	[M + H]^+^	680.1583	681.1656	681.1674	2.6	301, 531	MS3*, BS2
**Flavonols**										
16	3‐Methoxynobiletin	C_22_H_24_O_9_	56.651	**[M + H]^+^	432.1446	433.1519	433.1512	−1.6	403, 385, 373, 345	KS1*, BS1, DNS1, HS1, HS3
**Isoflavonoids**										
17	3’‐Hydroxydaidzein	C_15_H_10_O_5_	7.841	[M + H]^+^	270.0509	271.0582	271.0585	1.1	253, 241, 225	HS1*, BS1
18	5,6,7,3′,4’‐Pentahydroxyisoflavone	C_15_H_10_O_7_	12.466	[M + H]^+^	302.041	303.0483	303.0491	2.6	285, 257	MS3*
19	6”‐*O*‐Malonylgenistin	C_24_H_22_O_13_	55.864	[M + H]^+^	518.1087	519.1160	519.1145	−2.9	271	MS2*, BS1, BS2, HS2, TS2, DNS2, KS3
20	2′,7‐Dihydroxy‐4′,5′‐dimethoxyisoflavone	C_17_H_14_O_6_	65.442	[M + H]^+^	314.0808	315.0881	315.0878	−1.0	300, 282	BS1*, KS1, TS2
21	6”‐O‐Malonyldaidzin	C_24_H_22_O_12_	66.150	[M + H]^+^	502.1102	503.1175	503.1183	1.6	255	MS3*
**Other phenolic compounds** **Alkylmethoxyphenols**										
22	4‐Vinylsyringol	C_15_H_14_O_3_	56.135	[M + H]^+^	242.0965	243.1038	243.1030	−3.3	225, 211, 197	DNS3*, HS1, KS2, MS2
**Curcuminoids**										
23	Bisdemethoxycurcumin	C_19_H_16_O_4_	25.928	[M + H]^+^	308.1044	309.1117	309.1125	2.6	291, 263	TS2*
**Stilbenes**										
24	3’‐Hydroxy‐3,4,5,4′‐tetramethoxystilbene	C_17_H_18_O_5_	58.472	[M + H]^+^	302.1128	303.1201	303.1210	3.0	229, 201, 187, 175	BS1*, KS1, MS2
25	4‐Hydroxy‐3,5,4′‐trimethoxystilbene	C_17_H_18_O_4_	59.370	[M + H]^+^	286.1211	287.1284	287.1292	2.8	271, 241, 225	KS2*, BS2, DNS2
**Lignans**										
26	Conidendrin	C_20_H_20_O_6_	56.801	[M + H]^+^	356.1276	357.1349	357.1358	2.5	339, 221, 206	MS1*, HS1, BS2, HS3, MS3
27	Enterolactone	C_18_H_18_O_4_	56.839	[M + H]^+^	298.1219	299.1292	299.1288	−1.3	281, 187, 165	HS2*
28	7‐Oxomatairesinol	C_20_H_20_O_7_	63.957	[M + H]^+^	372.1208	373.1281	373.1271	−2.7	358, 343, 328, 325	KS3*, MS3, DNS3

* Compound was detected in more than one sample, and the data presented in this table are from asterisk sample.

** Compounds were detected in both negative [M‐H]^−^ and positive [M + H]^+^ mode of ionization while only single mode data was presented. Abbreviations were defined in Figure 1.

#### 3.4.1 Phenolic Acids

The 4 identified phenolic acids included 2 hydroxybenzoic acids, 1 hydroxycinnamic acid, and 1 hydroxyphenylpentanoic acid.

##### 3.4.1.1 Hydroxybenzoic Acids

Compound **1**, tentatively identified as 3,4‐O‐Dimethylgallic acid, was only detected in positive ionization mode in sample HS2. This compound was also identified in both positive and negative modes, with an existing [M + H]^+^ *m/z* of 199.0609. This compound has been previously identified in fermented papaya extracts (Yücetepe et al. 2021). Another hydroxybenzoic acid (Compound **2**) was tentatively identified as 4‐*O*‐Methylgallic acid (retention time = 51.955 min, *m/z* 185.0422) in MS2, DNS1, KS2, BS2, and DNS2 samples. This identification was further supported by the presence of product ions at *m/z* 170 and 142. 4‐O‐Methylgallic acid has been previously detected in mangoes and their by‐products (Dorta et al. 2014).

##### 3.4.1.2 Hydroxycinnamic Acids

Only one hydroxycinnamic acid was identified in this study, specifically from the *Khalal* and *Tamar* stages of the Thoory cultivar. Compound **3** (retention time = 56.910 min and an *m/z* 387.1066) was tentatively identified as 5–5’‐Dehydrodiferulic acid and further confirmed by the presence of a product ion at *m/z* 369.

##### 3.4.1.3 Hydroxyphenylpentanoic Acids

One hydroxyphenylpentanoic acid was identified in positive mode in samples HS1 and KS2. Compound **4** was tentatively identified as 5‐(3′‐Methoxy‐4′‐hydroxyphenyl)‐γ‐valerolactone, with the precursor ion observed at *m/z* 223.0957 and was further confirmed by the product ion at *m/z* 205 in MS/MS analysis.

#### 3.4.2 Flavonoids

The main phenolic compounds identified were flavonoid conjugates. The 17 identified flavonoids included 6 subtypes: 2 anthocyanin, 1 dihydrochalcone, 2 flavanols, 1 flavanone, 5 flavones, 1 flavonol, and 5 isoflavonoids. In particular, isoflavonoids and flavones were the most abundant subtypes.

##### 3.4.2.1 Anthocyanins

Two anthocyanins were identified in this study, tentatively identified as 4‐*O*‐Methyldelphinidin 3‐*O*‐D‐glucoside (Compound **5**) and Cyanidin 3‐*O*‐(2‐*O*‐(6‐*O*‐(E)‐caffeoyl‐D glucoside)‐D‐glucoside)‐5‐*O*‐D‐glucoside (Compound **6**). 4‐*O*‐Methyldelphinidin 3‐*O*‐D‐glucoside was found only in the Thoory cultivar at the *Kimri* stage. Cyanidin 3‐*O*‐(2‐*O*‐(6‐*O*‐(E)‐caffeoyl‐D glucoside)‐D‐glucoside)‐5‐*O*‐D‐glucoside was tentatively identified in DNS2 with a precursor ion at *m/z* 950.2677 and a retention time of 62.291 min.

##### 3.4.2.2 Dihydrochalcones

Only one dihydrochalcone was identified in this study. Compound **7**, tentatively identified as 3‐Hydroxyphloretin 2'‐O‐xylosyl‐glucoside, was detected in samples TS2, KS1, and TS3. This identification was based on the [M‐H]^−^ ion at *m/z* 583.1697 and the MS/MS product ion at m/z 289, which resulted from the loss of a xylosyl‐glucoside disaccharide (Pignatelli et al. 2000).

##### 3.4.2.3 Flavanols

Compound **8**, tentatively identified as 4'‐O‐Methylepigallocatechin 3‐O‐gallate, was detected in samples HS2, TS1, KS2, and BS2, with a precursor ion at *m/z* 321.0974 and product ions at *m/z* 319 and 169. Compound **9**, tentatively identified as 4'‐O‐methylepigallocatechin, was detected in positive ionization mode in DNS3 sample, with a precursor ion [M + H]^+^ at *m/z* 341.1373. This compound has been previously identified in *Elaeodendron transvaalense*, a southern African plant, as reported by Khumalo et al. (2019).

##### 3.4.2.4 Flavanones

Compound **10** was tentatively identified as 8‐Prenylnaringenin (retention time = 57.621 min with *m/z* 341.1373) in positive mode of sample HS3. This compound (8‐Prenylnaringenin) is primarily found in hops and has also been reported in *Moringa oleifera* pods (Xie et al. 2024). This compound has demonstrated significant potential as a phytoestrogen and has been shown to induce biological effects (Pohjanvirta and Nasri 2022).

##### 3.4.2.5 Flavones

Flavones were among the predominant group of flavonoids identified in the date fruit samples, comprising a total of 5 compounds. Compound **11** was tentatively identified as Apigenin 7‐*O*‐apiosyl‐glucoside in positive mode, as evidenced by the observed [M + H]^+^ at *m/z* 565.1548. This identification was further confirmed by the presence of the product ion at *m/z* 296. Compound **13**, tentatively identified as Luteolin 7‐O‐(2‐apiosyl‐glucoside), was observed in 10 date fruit samples across all three ripening stages (five from *Kimri* stage, two from *Khalal* stage and three from *Tamar* stage). Thoory was the only cultivar where Compound **13** was detected at all three ripening stages. Compound **14** (retention time = 58.839 min with *m/z* 359.1132) was tentatively identified as Gardenin B. This compound, previously identified in peppermint leaves, has demonstrated antiviral properties and is considered a promising candidate for the development of antiviral therapies (Al‐Karmalawy et al. 2021; Areias et al. 2001).

##### 3.4.2.6 Flavonols

Compound **16** was tentatively identified as 3‐Methoxynobiletin at both modes and observed in samples KS1, BS1, DNS1, HS1, and HS3, as evidenced by the observed [M + H]^+^ *m/z* value of 433.1512. This identification was further verified by the produced product ion at *m/z* 403, 385, 373, and 345. This compound was predominantly found at the *Kimri* stage and decreased in abundance as the fruit matured.

##### 3.4.2.7 Isoflavonoids

There were two unique compounds (Compounds **18** and **21**) observed only in sample MS3. At positive mode, Compound **18** was tentatively identified as 5,6,7,3′,4’‐Pentahydroxyisoflavone due to the retention time at 12.466 min and the observed [M + H]^+^ *m/z* value of 303.0491. Compound **19** was identified tentatively as 6”‐O‐Malonyldaidzin due to the retention time at 66.150 min and the observed [M + H]^+^ *m/z* value of 503.1183. Compound **19 (**retention time = 55.863 min with *m/z* 519.1145) was tentatively identified as 6”‐O‐Malonylgenistin. This compound was detected in samples MS2, BS1, BS2, HS2, TS2, DNS2, and KS3. 6”‐O‐Malonylgenistin mainly existed in date fruits at the *Khalal* stage (MS2, BS2, HS2, TS2, and DNS2) but all disappeared from these cultivars after the *Tamar* stage. Compound **20** was identified tentatively as 2′,7‐dihydroxy‐4′,5′‐dimethoxyisoflavone using a positive ion mode, as indicated by an *m/z* of 315.0878 and a retention time of 65.442 min. This compound, previously reported in *Lepidium sativum* seedcake by Kadam et al. (2018), was detected in Barhee and Khadrawy cultivars at *Kimri* stage and Thoory cultivar in *Khalal* stage.

#### 3.4.3 Other Phenolic Compounds

Other phenolic compounds included one Alkylmethoxyphenol, one Curcuminoid, two Stilbenes, and three Lignans. Compound **22**, tentatively identified as 4‐Vinylsyringol, was detected in samples DNS3, HS1, KS2, and MS2 with an [M + H]^+^ ion at m/z 243.103. Compound **23**, tentatively identified as Bisdemethoxycurcumin, was detected in sample TS2 with an [M + H]^+^ ion at *m/z* 309.1125.

##### 3.4.3.1 Stilbenes

Two stilbene compounds were identified: 3’‐Hydroxy‐3,4,5,4′‐tetramethoxystilbene (Compound **24**), detected in samples BS1, KS1, and MS2 with a retention time of 58.472 min and an *m/z* of 303.1210, and 4‐Hydroxy‐3,5,4′‐trimethoxystilbene (Compound **25**), detected in positive mode at *m/z* 287.1292 in samples KS2, BS2, and DNS2 (all at the *Khalal* stage).

##### 3.4.3.2 Lignans

Three lignan compounds were identified: Conidendrin (Compound **26**), detected in positive mode at *m/z* 357.1358 in multiple samples; Enterolactone (Compound **27**), detected at *m/z* 299.1288 in sample HS2; and 7‐Oxomatairesinol (Compound **28**), detected at *m/z* 373.1271 in samples KS3, MS3, and DNS3.

### 3.5 Distribution of Phenolic Compounds From LC–MS

**FIGURE 3:**
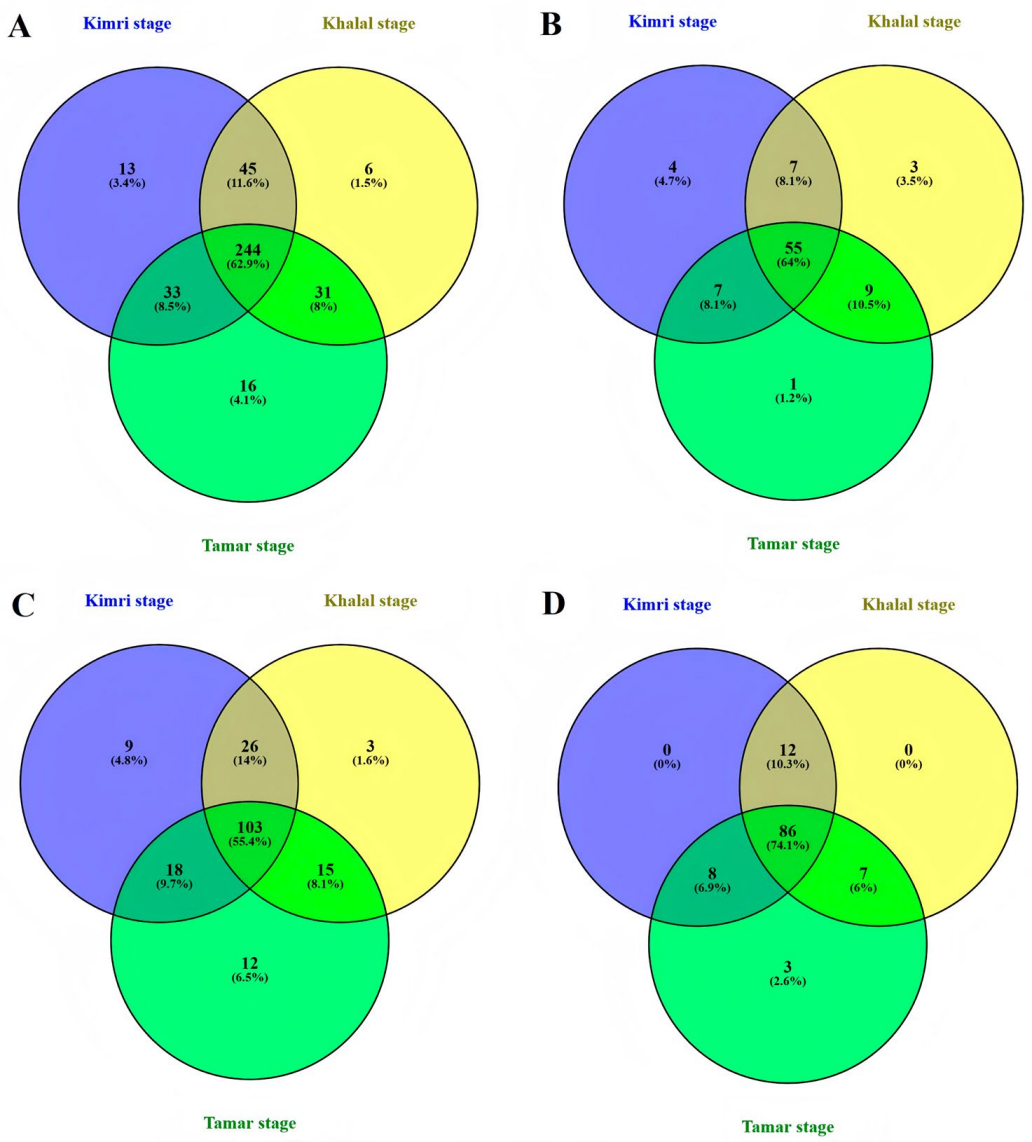
Venn diagram of phenolic compounds in date fruit cultivars presented at different ripening stages. (A) shows the comparison of total phenolic compounds at different ripening stages. (B) shows the comparison of total phenolic acids in different ripening stages. (C) shows the comparison of flavonoids in different ripening stages. (D) shows the comparison of other polyphenol compounds in different ripening stages.

Venn diagrams were used to visualize the overlap between different phenolic compound types and ripening stages (Figure 3). A total of 388 phenolic compounds were identified across all date samples (Figure 3A). A total of 244 (62.9%) of these compounds were shared across all ripening stages. The *Tamar* stage had the highest number of unique compounds (16), but the overall number of phenolic compounds was lowest at this stage.

Figure 3B shows that 86 phenolic acids were identified across all ripening stages, with the *Kimri* stage having the highest number of unique acids (4.7%), followed by *Khalal* and *Tamar*. Similarly, Figure 3C shows 186 flavonoids across the three ripening stages, with *Tamar* having the highest number of unique flavonoids (12% or 6.5%). Figure 3D shows 86 other phenolic compounds, with 74.1% shared across all ripening stages. Dates at the *Kimri* and *Khalal* stages had no unique phenolic compounds, while Tamar had 3 (2.6%). Overall, a decreasing trend in phenolic compound abundance was observed with fruit ripening, similar to the trends seen for phenolic acids and flavonoids.

Flavonoids were the most abundant phenolic compounds, with 186 identified, followed by phenolic acids (86) and other phenolic compounds (116). A general trend of decreasing phenolic compounds was observed with fruit ripening. This agrees with previous studies suggesting enzymatic activity reduction with ripening (Bano et al. 2022).

### 3.6 Heatmap and Hierarchical Clustering Analysis

**FIGURE 4:**
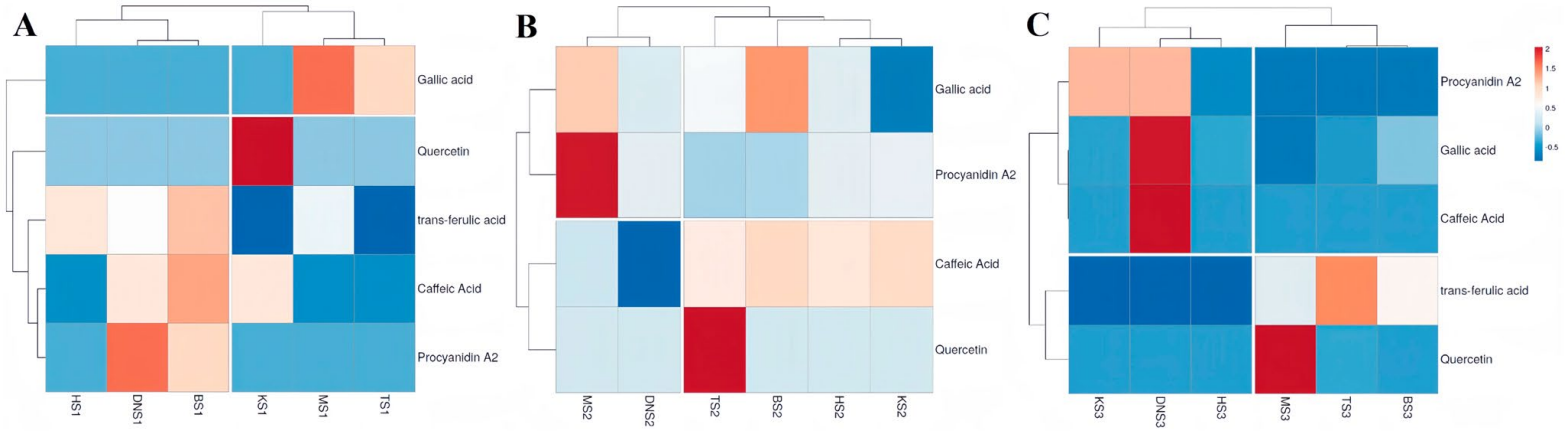
Heatmap showing distribution and concentration of phenolic compounds across six date fruit cultivars at *Kimri* (A), *Khalal* (B), and *Tamar* stages (C). Red boxes indicate higher concentration. Blue boxes indicate lower concentration.

A heatmap was generated to visualize the hierarchical clustering of phenolic compounds analyzed by LC‐DAD in 18 date fruit samples (Figure 4). The heatmap illustrates the correlation between samples and compounds, with rows and columns clustered based on the average concentration of each compound. The most closely related samples and compounds were grouped together.

Figure 4A shows the phenolic content in date fruit at the *Kimri* stage, with the Khadrawy cultivar having a high quercetin concentration at this stage, approximately twice the concentration of other cultivars. The Mejhoul cultivar had the highest gallic acid content, but the Deglet nour cultivar had the highest Procyanidin A2 content. The Barhee cultivar had a unique phenolic profile, with constituents such as trans‐ferulic acid, caffeic acid, and procyanidin A2 registering concentrations approximately 1.5 times higher than the average concentration, highlighting the distinct phenolic profile of this cultivar. As shown in Figure 4B, the Thoory cultivar at the *Khalal* stage had a significantly higher concentration of quercetin. The Mejhoul cultivar also had a high content of Procyanidin A2, approximately 2 times the average level. The Halawi date cultivar had a higher concentration of gallic acid, about 1.5 times higher than the average concentration found in other cultivars of dates. At the *Tamar* stage (Figure 4C), there was a rapid change in the phenolic compound content across cultivars. Most phenolic compounds decreased significantly at the *Tamar* stage. However, the Deglet nour maintained a high concentration of Procyanidin A2, gallic acid, and caffeic acid. The Khadrawy cultivar also had a high Procyanidin A2 concentration, while the Mejhoul had the highest quercetin content. The Thoory cultivar maintained a high trans‐ferulic acid concentration at this ripening stage.

## 4 Conclusions

This study investigated the phenolic composition and antioxidant properties of six Australian date palm cultivars at three ripening stages. LC‐ESI‐QTOF‐MS/MS analysis identified 28 phenolic compounds, many of which are known for their health benefits. Our findings suggest a general decline in antioxidant capacity and phenolic content as date fruits ripen. Date fruits at the initial stages of development, particularly from Thoory and Deglet nour cultivars, could serve as a rich source of natural antioxidants. Future research should investigate the potential of these fruits as functional food ingredients or nutraceutical supplements, promoting sustainable utilization of discarded fruit from early ripening stages.

## Author Contributions

**Linghong Shi:** conceptualization (equal), formal analysis (equal), investigation (equal), methodology (equal), software (equal), writing – original draft (equal). **Manan Sejpal:** methodology (equal), writing – review and editing (equal). **Kashif Ghafoor:** supervision (equal), writing – review and editing (equal). **Claudia Gonzalez Viejo:** supervision (equal), writing – review and editing (equal). **Sigfredo Augusto Fuentes Jara:** supervision (equal), writing – review and editing (equal). **Farhad Ahmadi:** data curation (equal), supervision (equal), validation (equal), visualization (equal), writing – review and editing (equal). **Hafiz A. R. Suleria:** conceptualization (equal), funding acquisition (equal), investigation (equal), methodology (equal), project administration (equal), resources (equal), supervision (equal), validation (equal), visualization (equal), writing – review and editing (equal).

## Conflicts of Interest

The authors declare no conflicts of interest.

## Data Availability

The data that support the findings of this study are available from the corresponding author upon reasonable request.

## References

[fsn370221-bib-0001] AlFaris, N. A. , J. Z. AlTamimi , F. A. AlGhamdi , et al. 2021. “Total Phenolic Content in Ripe Date Fruits ( *Phoenix dactylifera* L.): A Systematic Review and Meta‐Analysis.” Saudi Journal of Biological Sciences 28, no. 6: 3566–3577. 10.1016/j.sjbs.2021.03.033. 34121900 PMC8175999

[fsn370221-bib-0002] Al‐Karmalawy, A. A. , M. M. Farid , A. Mostafa , et al. 2021. “Naturally Available Flavonoid Aglycones as Potential Antiviral Drug Candidates Against SARS‐CoV‐2.” Molecules 26, no. 21: 6559. 34770969 10.3390/molecules26216559 PMC8587465

[fsn370221-bib-0003] Amira, E. A. , S. E. Behija , M. Beligh , et al. 2012. “Effects of the Ripening Stage on Phenolic Profile, Phytochemical Composition and Antioxidant Activity of Date Palm Fruit.” Journal of Agricultural and Food Chemistry 60, no. 44: 10896–10902. 10.1021/jf302602v. 23072597

[fsn370221-bib-0004] Areias, F. M. , P. Valentão , P. B. Andrade , F. Ferreres , and R. M. Seabra . 2001. “Phenolic Fingerprint of Peppermint Leaves.” Food Chemistry 73, no. 3: 307–311. 10.1016/S0308-8146(00)00302-2.

[fsn370221-bib-0005] Bano, Y. , A. Rakha , M. I. Khan , and M. Asgher . 2022. “Chemical Composition and Antioxidant Activity of Date (*Phoenix dactylifera* L.) Varieties at Various Maturity Stages.” Food Science and Technology 42: e29022. 10.1590/fst.29022.

[fsn370221-bib-0006] Blois, M. S. 1958. “Antioxidant Determinations by the Use of a Stable Free Radical.” Nature 181, no. 4617: 1199–1200.

[fsn370221-bib-0007] Christ, B. , and K. Müller . 1960. “Zur Serienmäßigen Bestimmung des Gehaltes an Flavonol‐Derivaten in Drogen.” Archiv der Pharmazie 293, no. 12: 1033–1042. 10.1002/ardp.19602931202. 13693338

[fsn370221-bib-0008] Dhara, A. K. , and A. K. Nayak . 2022. “Chapter 1 ‐ Introduction to Herbal Biomolecules.” In Herbal Biomolecules in Healthcare Applications, edited by S. C. Mandal , A. K. Nayak , and A. K. Dhara , 1–19. Academic Press.

[fsn370221-bib-0009] Dinis, T. C. P. , V. M. C. Madeira , and L. M. Almeida . 1994. “Action of Phenolic Derivatives (Acetaminophen, Salicylate, and 5‐Aminosalicylate) as Inhibitors of Membrane Lipid Peroxidation and as Peroxyl Radical Scavengers.” Archives of Biochemistry and Biophysics 315, no. 1: 161–169. 10.1006/abbi.1994.1485. 7979394

[fsn370221-bib-0010] Dorta, E. , M. González , M. G. Lobo , C. Sánchez‐Moreno , and B. de Ancos . 2014. “Screening of Phenolic Compounds in By‐Product Extracts From Mangoes ( *Mangifera indica* L.) by HPLC‐ESI‐QTOF‐MS and Multivariate Analysis for Use as a Food Ingredient.” Food Research International 57: 51–60.

[fsn370221-bib-0012] Haider, M. S. , I. A. Khan , M. J. Jaskani , et al. 2018. “Pomological and Biochemical Profiling of Date Fruits (*Phoenix dactylifera* L.) During Different Fruit Maturation Phases.” Pakistan Journal of Botany 50, no. 3: 1069–1076.

[fsn370221-bib-0013] Jdaini, K. , F. Alla , F. Mansouri , A. Parmar , and M. A. Elhoumaizi . 2023. “Optimizing the Extraction of Phenolic Antioxidants From Date Palm Fruit by Simplex‐Centroid Solvent Mixture Design.” Heliyon 9, no. 1: e12738. 36685389 10.1016/j.heliyon.2022.e12738 PMC9852673

[fsn370221-bib-0014] Kadam, D. , S. Palamthodi , and S. Lele . 2018. “LC–ESI‐Q‐TOF–MS/MS Profiling and Antioxidant Activity of Phenolics From *L. Sativum* Seedcake.” Journal of Food Science and Technology 55, no. 3: 1154–1163. 10.1007/s13197-017-3031-8. 29487458 PMC5821675

[fsn370221-bib-0015] Khatib, M. , A. Al‐Tamimi , L. Cecchi , et al. 2022. “Phenolic Compounds and Polysaccharides in the Date Fruit ( *Phoenix dactylifera* L.): Comparative Study on Five Widely Consumed Arabian Varieties.” Food Chemistry 395: 133591. 10.1016/j.foodchem.2022.133591. 35780667

[fsn370221-bib-0016] Khumalo, G. , N. Sadgrove , S. Van Vuuren , and B.‐E. Van Wyk . 2019. “Antimicrobial Lupenol Triterpenes and a Polyphenol From Elaeodendron Transvaalense, a Popular Southern African Medicinal Bark.” South African Journal of Botany 122: 518–521. 10.1016/j.sajb.2018.07.020.

[fsn370221-bib-0017] Kutlu, T. , K. Takim , B. Çeken , and M. Kizil . 2014. “DNA Damage Protecting Activity and In Vitro Antioxidant Potential of the Methanol Extract of Cherry (*Prunus avium* L).” Journal of Medicinal Plant Research 8, no. 19: 715–726.

[fsn370221-bib-0018] Mohamed Lemine, F. M. , M. V. Mohamed Ahmed , L. Ben Mohamed Maoulainine , A. Bouna Zel , A. Samb , and O. B. AO . 2014. “Antioxidant Activity of Various Mauritanian Date Palm (*Phoenix dactylifera* L.) Fruits at Two Edible Ripening Stages.” Food Science & Nutrition 2, no. 6: 700–705. 10.1002/fsn3.167. 25493188 PMC4256575

[fsn370221-bib-0019] Mohamed, R. M. A. , A. S. M. Fageer , M. M. Eltayeb , and I. A. Mohamed Ahmed . 2014. “Chemical Composition, Antioxidant Capacity, and Mineral Extractability of Sudanese Date Palm ( *Phoenix dactylifera* L.) Fruits.” Food Science & Nutrition 2, no. 5: 478–489. 10.1002/fsn3.123. 25473506 PMC4237478

[fsn370221-bib-0020] Oyaizu, M. 1986. “Studies on Products of Browning Reaction Antioxidative Activities of Products of Browning Reaction Prepared From Glucosamine.” Japanese Journal of Nutrition and Dietetics 44, no. 6: 307–315.

[fsn370221-bib-0021] Pignatelli, P. , F. M. Pulcinelli , A. Celestini , et al. 2000. “The Flavonoids Quercetin and Catechin Synergistically Inhibit Platelet Function by Antagonizing the Intracellular Production of Hydrogen Peroxide.” American Journal of Clinical Nutrition 72, no. 5: 1150–1155. 11063442 10.1093/ajcn/72.5.1150

[fsn370221-bib-0022] Pohjanvirta, R. , and A. Nasri . 2022. “The Potent Phytoestrogen 8‐Prenylnaringenin: A Friend or a Foe?” International Journal of Molecular Sciences 23, no. 6: 3168. 10.3390/ijms23063168. 35328588 PMC8953904

[fsn370221-bib-0023] Price, M. L. , S. Van Scoyoc , and L. G. Butler . 1978. “A Critical Evaluation of the Vanillin Reaction as an Assay for Tannin in Sorghum Grain.” Journal of Agricultural and Food Chemistry 26, no. 5: 1214–1218.

[fsn370221-bib-0024] Prieto, P. , M. Pineda , and M. Aguilar . 1999. “Spectrophotometric Quantitation of Antioxidant Capacity Through the Formation of a Phosphomolybdenum Complex: Specific Application to the Determination of Vitamin E.” Analytical Biochemistry 269, no. 2: 337–341. 10222007 10.1006/abio.1999.4019

[fsn370221-bib-0025] Rashidinejad, A. , and M. K. Ahmmed . 2024. “The Influence of Ripening on the Nutrient Composition and Antioxidant Properties of New Zealand Damson Plums.” Food Science & Nutrition 12, no. 6: 4311–4320. 10.1002/fsn3.4097. 38873447 PMC11167141

[fsn370221-bib-0026] Re, R. , N. Pellegrini , A. Proteggente , A. Pannala , M. Yang , and C. Rice‐Evans . 1999. “Antioxidant Activity Applying an Improved ABTS Radical Cation Decolorization Assay.” Free Radical Biology and Medicine 26, no. 9–10: 1231–1237. 10381194 10.1016/s0891-5849(98)00315-3

[fsn370221-bib-0027] Salgado, P. , V. Melin , D. Contreras , Y. Moreno , and H. D. Mansilla . 2013. “Fenton Reaction Driven by Iron Ligands.” Journal of the Chilean Chemical Society 58, no. 4: 2096–2101.

[fsn370221-bib-0028] Shahdadi, F. , H. O. Mirzaei , and A. Daraei Garmakhany . 2015. “Study of Phenolic Compound and Antioxidant Activity of Date Fruit as a Function of Ripening Stages and Drying Process.” Journal of Food Science and Technology 52, no. 3: 1814–1819. 10.1007/s13197-013-1177-6. 25745262 PMC4348321

[fsn370221-bib-0029] Shi, L. , W. Li , M. S. Rahman , et al. 2023. “Comparison of Phenolic Composition in Date (*Phoenix dactylifera* L.) Flesh and Seeds Extracted by an Ultrasonic‐Assisted and Conventional Method.” International Journal of Food Properties 26, no. 2: 2939–2962. 10.1080/10942912.2023.2261787.

[fsn370221-bib-0030] Shi, L. , Z. Liu , C. Gonzalez Viejo , F. Ahmadi , F. R. Dunshea , and H. A. R. Suleria . 2024. “Comparison of Phenolic Composition in Australian‐Grown Date Fruit ( *Phoenix dactylifera* L.) Seeds From Different Varieties and Ripening Stages.” Food Research International 181: 114096. 10.1016/j.foodres.2024.114096. 38448106

[fsn370221-bib-0031] Slinkard, K. , and V. L. Singleton . 1977. “Total Phenol Analysis: Automation and Comparison With Manual Methods.” American Journal of Enology and Viticulture 28, no. 1: 49–55.

[fsn370221-bib-0032] Socaci, S. A. , D. O. Rugină , Z. M. Diaconeasa , et al. 2017. “Antioxidant Compounds Recovered From Food Wastes.” Functional Food‐Improve Health Through Adequate Food: 3–22. Intechopen.

[fsn370221-bib-0033] Sogi, D. S. , M. Siddiq , I. Greiby , and K. D. Dolan . 2013. “Total Phenolics, Antioxidant Activity, and Functional Properties of ‘Tommy Atkins’ Mango Peel and Kernel as Affected by Drying Methods.” Food Chemistry 141, no. 3: 2649–2655. 23871007 10.1016/j.foodchem.2013.05.053

[fsn370221-bib-0034] Souli, I. , M. Jemni , L. L. Rodríguez‐Verástegui , N. Chaira , F. Artés , and A. Ferchichi . 2018. “Phenolic Composition Profiling of Tunisian 10 Varieties of Common Dates (*Phoenix dactylifera* L.) at Tamar Stage Using LC‐ESI‐MS and Antioxidant Activity.” Journal of Food Biochemistry 42, no. 6: e12634. 10.1111/jfbc.12634.

[fsn370221-bib-0035] Suleria, H. A. , C. J. Barrow , and F. R. Dunshea . 2020. “Screening and Characterization of Phenolic Compounds and Their Antioxidant Capacity in Different Fruit Peels.” Food 9, no. 9: 1206. 10.3390/foods9091206. PMC7556026 32882848

[fsn370221-bib-0036] Uwineza, P. A. , and A. Waśkiewicz . 2020. “Recent Advances in Supercritical Fluid Extraction of Natural Bioactive Compounds From Natural Plant Materials.” Molecules 25, no. 17: 3847. 32847101 10.3390/molecules25173847 PMC7504334

[fsn370221-bib-0037] Xie, R. , E. N. Ponnampalam , F. Ahmadi , F. R. Dunshea , and H. A. R. Suleria . 2024. “Antioxidant Potential and Characterization of Polyphenol Compounds in Pods.” Food Science & Nutrition 12: 10881–10902. 10.1002/fsn3.4628. 39723086 PMC11666903

[fsn370221-bib-0038] Yücetepe, A. , G. Altin , and B. Özçelik . 2021. “A Novel Antioxidant Source: Evaluation of In Vitro Bioaccessibility, Antioxidant Activity and Polyphenol Profile of Phenolic Extract From Black Radish Peel Wastes (*Raphanus sativus* L. var. Niger) During Simulated Gastrointestinal Digestion.” International Journal of Food Science & Technology 56, no. 3: 1376–1384. 10.1111/ijfs.14494.

[fsn370221-bib-0039] Zhang, C.‐R. , S. A. Aldosari , P. S. Vidyasagar , K. M. Nair , and M. G. Nair . 2013. “Antioxidant and Anti‐Inflammatory Assays Confirm Bioactive Compounds in Ajwa Date Fruit.” Journal of Agricultural and Food Chemistry 61, no. 24: 5834–5840. 10.1021/jf401371v. 23713661

[fsn370221-bib-0040] Zihad, S. M. N. K. , S. J. Uddin , N. Sifat , et al. 2021. “Antioxidant Properties and Phenolic Profiling by UPLC‐QTOF‐MS of Ajwah, Safawy and Sukkari Cultivars of Date Palm.” Biochemistry and Biophysics Reports 25: 100909. 10.1016/j.bbrep.2021.100909. 33521336 PMC7820033

